# Infectious Morbidity After Radical Vulvectomy

**DOI:** 10.1155/S1064744994000529

**Published:** 1994

**Authors:** Steven A. Elg, Linda F. Carson, Doris C. Brooker, Jonathan R. Carter, Leo B. Twiggs

**Affiliations:** 1 Department of Obstetrics and Gynecology Tripler Army Medical Center Tripler AMC HI 96859-5000 USA; 2 Department of Obstetrics and Gynecology University of Minnesota Minneapolis MN USA

## Abstract

*Objective:* This retrospective investigation describes the infectious morbidity of patients following radical vulvectomy with or without inguinal lymph node dissection.

*Methods:* The charts of patients undergoing radical vulvectomy between January 1, 1986, and September 1, 1989, were reviewed for age, weight, cancer type, tumor stage, operative procedure(s), prophylactic antibiotic and its length of use, febrile morbidity, infection site, culture results, significant medical history, and length of use and number of drains or catheters used.

*Results:* The study group was composed of 61 patients, 14 of whom underwent a radical vulvectomy and 47 who also had inguinal lymph node dissection performed. Twenty-nine patients (48%) had at least 1 postoperative infection. Five patients (8%) had 2 or more postoperative infections. The site and incidence of the infections were as follows: urinary tract 23%, wound 23%, lymphocyst 3%, lymphatics (lymphangitis) 5%, and bowel (pseudomembranous colitis) 3%. The most common pathogens isolated from both urine and wound sites were *Pseudomonas aeruginosa*, enterococcus, and *Escherichia coli.* A significant decrease in wound infection was demonstrated when separate incisions were made for inguinal lymph node dissection (*P* <0.05). The mean number of days to onset of postoperative infection for wound, urine, lymphatics, lymphocyst, and bowel were 11, 8, 57, 48, and 5, respectively.

*Conclusions:* We conclude that the clinical appearance of post-radical vulvectomy infections is delayed when compared with other post-surgical wound infections. Second, utilizing separate inguinal surgical incisions may reduce infectious morbidity. Finally, tumor stage and type do not necessarily increase the infectious morbidity of radical vulvar surgery.


Infectious Diseases in Obstetrics and Gynecology 2:130–135 (1994)
© 1994 Wiley-Liss, Inc.

### Infectious Morbidity After Radical Vulvectomy

**Steven A. Elg, Linda F. Carson, Doris C. Brooker, Jonathan R. Carter, and Leo B. Twiggs**

*Department of Obstetrics and Gynecology, Tripler Army Medical Center, Tripler AMC, HI (S.A.E.), and Department of Obstetrics and Gynecology, University of Minnesota, Minneapolis, MN (L.F.C., D.C.B., J.R.C., L.B.T.)*

### Abstract

***Objective:*** **This retrospective investigation describes the infectious morbidity of patients following radical vulvectomy with or without inguinal lymph node dissection.**

***Methods:*** **The charts of patients undergoing radical vulvectomy between January 1, 1986, and September 1, 1989, were reviewed for age, weight, cancer type, tumor stage, operative procedure(s), prophylactic antibiotic and its length of use, febrile morbidity, infection site, culture results, significant medical history, and length of use and number of drains or catheters used.**

***Results:*** **The study group was composed of 61 patients, 14 of whom underwent a radical vulvectomy and 47 who also had inguinal lymph node dissection performed. Twenty-nine patients (48%) had at least 1 postoperative infection. Five patients (8%) had 2 or more postoperative infections. The site and incidence of the infections were as follows: urinary tract 23%, wound 23%, lymphocyst 3%, lymphatics (lymphangitis) 5%, and bowel (pseudomembranous colitis) 3%. The most common pathogens isolated from both urine and wound sites were *Pseudomonas aeruginosa*, enterococcus, and *Escherichia coli*. A significant decrease in wound infection was demonstrated when separate incisions were made for inguinal lymph node dissection ($P < 0.05$). The mean number of days to onset of postoperative infection for wound, urine, lymphatics, lymphocyst, and bowel were 11, 8, 57, 48, and 5, respectively.**

***Conclusions:*** **We conclude that the clinical appearance of post-radical vulvectomy infections is delayed when compared with other post-surgical wound infections. Second, utilizing separate inguinal surgical incisions may reduce infectious morbidity. Finally, tumor stage and type do not necessarily increase the infectious morbidity of radical vulvar surgery.** © 1994 Wiley-Liss, Inc.

Key words

Vulvar cancer, surgical infections, inguinal lymph node dissection

Infectious morbidity/mortality in gynecologic oncology has not been the subject of extensive investigation. An understanding of the basis of these infections requires an understanding of the multiple infection risk factors in the host, those in the extended radical surgical procedures, and factors inherent in the tumor itself.[1,2]

Host factors include 1) normal vaginal flora; 2) altered flora and organism virulence in malignancy; 3) altered flora from previous use of antimicrobials including the induction of antimicrobial resistance and superinfection;[3–8] 4) general host defense factors such as mucosal barriers, cellular and humoral immunity, and normal and decreased neutrophil, lymphocyte, and macrophage function; 5) known surgical risk factors in women with malignancy such as older age, poor nutritional status (weight loss, obesity), and concurrent disease such

Address correspondence/reprint requests to Dr. Steven A. Elg, Department of Obstetrics and Gynecology, Tripler Army Medical Center, Tripler AMC, HI 96859-5000.

Received January 21, 1994
Accepted May 18, 1994

as diabetes, hypertension, and bacterial or viral sexually transmitted disease; and 6) previous chemotherapy and/or radiation therapy.

Risks derived from the radical surgical procedure include 1) prolonged time of operation with the increased dissection and tissue damage of the extended "radical" procedure and presence of blood and exudate in the operative site and 2) anesthesia risk.[10–12]

The major risks arising from the tumor itself are 1) preexisting subclinical infections in proliferating and necrotic malignancy and 2) possible immunosuppressive effects exerted by the tumor itself.[13–23]

In addition to the usual risks deriving from host factors, from surgical procedures, and from the tumor itself, invasive diagnostic techniques used before surgery add to the risk from the possibility of preexisting occult infection of the genital tissue and possible introduction of nosocomial infection. Invasive procedures such as cystoscopy, proctoscopy, needle biopsy, and dilatation and curettage are routine in the work-up of a patient with gynecologic malignancy.

Finally, modern supportive procedures that add to postoperative infection risk include ureteral catheterization, percutaneous nephrostomy, central venous catheterization, hyperalimentation, and use of Jackson-Pratt drainage tubes, as well as urethral catheterization, insertion of a nasogastric tube, and placement of an intravenous line, all of which are common in patients after surgery.[14,15]

Of the gynecologic malignancies, the literature is perhaps the most conspicuously lacking in information relating to infectious complications associated with vulvar cancer. Although wound breakdown has been reported to be as high as 50% in patients undergoing radical vulvectomy,[24] a description of the associated infectious morbidity has not been reported. The purpose of this retrospective study is to document specific elements of infectious morbidity in patients undergoing radical vulvectomy with or without inguinal lymph node dissection, namely, organisms encountered; site of infection; sequence and time course of the appearance of clinical infections; association with drains and catheters; effect of tumor type and stage; and influence of surgical incisions. This study does not replace the need for prospective randomized trials to evaluate prophylactic antibiotic use in these patients.

### SUBJECTS AND METHODS

The charts of all patients operated on for vulvar cancer at the University of Minnesota Women's Cancer Center between January 1, 1986, and September 1, 1989, were reviewed. Data retrieved included the patient's age, weight, cancer type, tumor stage, operative procedure(s), prophylactic antibiotic and its length of use, febrile morbidity, infection site, culture results, significant medical history, and length of use and number of drains or catheters. Patients receiving radiation or chemotherapy within 60 days of surgery were excluded from the study. Those with a history of radiation or vulvar surgery were also excluded.

The following criteria were used to retrospectively define infectious morbidity: 1) febrile morbidity—2 oral temperatures, at least 4 h apart, of 38°C or higher on a day beyond the 1st postoperative day or an oral temperature of 38.3°C or higher within the 1st 24 h of the postoperative period; 2) wound infection—distinct cellulitis (erythema, induration, and tenderness at the margin of the incision) and/or actual purulent drainage from the incision (simple stitch abscess, seroma, and hematoma were not considered true wound infections); 3) infected lymphocyst—induration, erythema, and/or tenderness overlying a lymphocyst in a febrile patient (with no other source of infection); 4) lymphangitis—induration, erythema, and tenderness extending down the lymphatics of the thigh or leg (in the opinion of the primary physician); 5) urinary tract infection (UTI)—urine culture obtained by catheterization, demonstrating at least $10^2$ uropathogens/ml of urine; 6) septicemia—positive aerobic or anaerobic blood culture in a febrile patient; and 7) pseudomembranous colitis—positive *Clostridium difficile* toxin or culture in the stool of a patient with diarrhea.

Comparisons between groups within the study population were made by means of the uncorrected chi-square test, the 2-tailed independent sample t-test, and the 2-way analysis of variance test. $P < 0.05$ was considered statistically significant.

### RESULTS

The study group was composed of 61 patients, 14 of whom underwent radical vulvectomy alone and 47 of whom had radical vulvectomy with inguinal lymph node dissection. Both same incision and separate incisions were utilized in inguinal lymph node

**TABLE 1. Frequency of tumor type and stage in infected and noninfected patients**

| Tumor type | | Stage | Noninfected | Infected |
|---|---|---|---|---|
| Squamous cell carcinoma | FIGO | I | 13 | 10 |
| | | II | 7 | 10 |
| | | III | 6 | 5 |
| | | Unknown | 1 | 1 |
| | | *Total* | *27* | *26* |
| Malignant melanoma | Clark's | III | 2 | 1 |
| | | IV | 0 | 1 |
| | | *Total* | *2* | *2* |
| Paget's disease | FIGO | IV | *0* | *1* |
| Adenocarcinoma of Bartholin's gland | FIGO | II | 1 | 0 |
| | | III | 1 | 0 |
| | | *Total* | *2* | *0* |
| Basal cell carcinoma | FIGO | II | *1* | *0* |
| Grand totals | | | *32* | *29* |

dissections. The type of incision made was dependent on surgeon preference and prior training and not on the extent of disease. No statistical difference in mean age (67 years) or weight (69 kg) was noted in the postoperative infected vs. noninfected group.

A frequency distribution of tumor type and stage in both the infected and noninfected groups of patients is given in Table 1. A significant difference in the frequency of infection with respect to tumor type and stage was not discernible ($P > 0.05$).

Twenty-nine patients (48%) had at least 1 postoperative infection. Excluding UTIs, 17 patients (28%) had at least 1 postoperative infection. Five patients (8%) had 2 or more postoperative infection sites. The incidence of infection at the different sites was as follows: urinary tract 23%, wound 23%, lymphocyst 3%, lymphatiso (lymphangitis) 5%, blood 0%, and bowel (pseudomembranous colitis) 3%.

Seven patients had a preoperative UTI at the time of admission. Organisms responsible were *Escherichia coli* in 4 patients, β-hemolytic streptococcus group C in 1 patient, *Pseudomonas aeruginosa* in 1 patient, and coagulase-negative staphylococcus in 1 patient. One patient with preoperative UTI with *E. coli* developed a postoperative UTI with *P. aeruginosa*. None of the other 6 patients had postoperative UTI.

Although we are unaware of the exact criteria used by the individual attending physicians to make the diagnosis of wound infection, using our definition we were retrospectively unable to identify any patients that went undiagnosed. There was evidence of erythema, induration, and tenderness of the incision margin in virtually all of these patients. It is more difficult to ascertain the incidence of purulent drainage from the wounds, since drainage was frequently mentioned but not well characterized. Two cases of wound abscess located in groin incisions were well documented.

**TABLE 2. Radical vulvectomy with inguinal lymphadenectomy, same incision vs. separate groin incisions**

| | Separate incisions | Same incision | |
|---|---|---|---|
| No. infected | 8 | 7[a] | |
| No. noninfected | 30 | 2 | |
| Total | 38 | 9 | ($P < 0.05$) |

[a]Five of these patients developed wound infections; 2 developed lymphangitis.

Patients undergoing radical vulvectomy had a 7% incidence of wound infection, while those undergoing concurrent inguinal lymph node dissection had an incidence of 28% ($P < 0.05$). Of note, patients undergoing radical vulvectomy with inguinal lymphadenectomy had significantly lower wound infections if separate incisions were used (Table 2). No difference in the incidence of UTI was noted in patients undergoing radival vulvectomy with or without inguinal lymph node dissection ($P > 0.05$).

The most common urinary pathogen isolated was *P. aeruginosa*. A complete list of organisms isolated from urine postoperatively is given in Table 3. The

**TABLE 3. Pathogens isolated from urine**

| Pathogen | No. instances isolated | No. instances isolated alone |
|---|---|---|
| *Pseudomonas aeruginosa* | 5 | 4 |
| *Escherichia coli* | 5 | 1 |
| Enterococcus | 3 | 3 |
| *Staphylococcus aureus* | 1 | 0 |
| *Enterobacter cloacae* | 1 | 0 |
| *Proteus mirabilis* | 1 | 1 |
| *Candida albicans* | 1 | 1 |

**TABLE 4. Pathogens isolated from wounds**

| Pathogen | No. instances isolated | No. instances isolated alone |
|---|---|---|
| Enterococcus | 6 | 0 |
| *Escherichia coli* | 3 | 1 |
| *Staphylococcus aureus* | 1 | 0 |
| *Pseudomonas aeruginosa* | 2 | 0 |
| *Enterobacter cloacae* | 2 | 0 |
| β-hemolytic streptococcus group G | 1 | 1 |
| α-hemolytic streptococcus | 2 | 1 |
| *Klebsiella oxytoca* | 1 | 0 |
| *Bacteroides uniformis* | 1 | 0 |
| *Proteus mirabilis* | 1 | 0 |
| *Klebsiella pneumoniae* | 1 | 0 |
| *Bacteroides* species | 1 | 0 |
| *Actinobacter calcoaceticus* | 1 | 0 |
| *Streptococcus viridans* | 1 | 0 |
| *Peptostreptococcus prevotti* | 1 | 0 |
| *Peptostreptococcus anaerobus* | 1 | 0 |
| *Eubacterium* species | 1 | 0 |

mean number of urethral catheter insertions per admission was 1.3 in both urinary-infected and noninfected patients. The mean duration of urethral drainage was 8.7 days in the infected group vs. 3.9 days in the noninfected group ($P < 0.05$).

Wound infections tended to be polymicrobial. As can be seen from Table 4, the most common organism isolated from infected wounds was enterococcus. The mean duration of Jackson-Pratt drainage in the wound infected vs. noninfected group was 9.9 and 9.5 days, respectively ($P > 0.05$). The amount of output per drain was not consistently retrievable from the charts.

*C. difficile* was isolated from the stool of 2 patients with diarrhea. One of these patients received only 1 dose of cefoxitin preoperatively while the other received 3 days of ampicillin and gentamicin. The 1st patient had also received trimethoprin-sulfamethoxazole the week prior to surgery for a presumed UTI.

The mean number of days to onset of postoperative infection for wound, urine, lymphatics, lymphocyst, and bowel were 11, 8, 57, 48, and 5, respectively.

The most common prophylactic antibiotic used was cefoxitin. It was used by itself in 22 cases and parenterally in combination with intraoperative cephalothin or cefazolin-irrigating solution in 8 cases. Other prophylactic antibiotics used alone or in combination included cefotetan, flagyl, trimethoprin-sulfamethoxazole, ampicillin, gentamicin, doxycycline, tetracycline, and erythromycin. Six patients did not receive any prophylactic antibiotic. Two of the patients had postoperative infections. *E. coli* was isolated from the urine of 1 patient while the other subsequently developed lymphangitis. Prophylatic antibiotic was administered from 1 to 3 days perioperatively. Due to the wide variation in the type, dosage, and duration of prophylactic antibiotic administered, the optimal type and length of prophylactic antibiotic use could not be determined.

One patient with advanced-stage vulvar cancer and no clinical evidence of infection had aerobic and anaerobic cultures of the necrotic tumor sent before surgery. Organisms recovered included *P. aeruginosa*, β-hemolytic streptococcus group B, anaerobic diptheroids, and anaerobic gram-negative cocci.

No organisms were isolated in the patients with lymphangitis, but 2 of 3 patients with this diagnosis had associated febrile morbidity. Drainage from 1 infected lymphocyst grew *Klebsiella pneumoniae* and that from another grew *Staphylococcus aureus*. Both patients with infected lymphocysts had associated fevers.

### DISCUSSION

To our knowledge, this is the first report looking specifically at infectious morbidity in patients undergoing radical vulvectomy. Three important observations were made in this study.

The first finding is the prolonged period of time until onset of infection. Whereas wound infection following hysterectomy or cesarean section tends to be within 7 days,[25–28] wound infection following radical vulvectomy averages 11 days. This relatively late onset of wound infection is surprising and makes us wonder if factors other than microbial contamination at the time of surgery might be the etiologic source. Perhaps presurgical translocation of bacteria from infected necrotic tumor sites or altered vaginal flora to regional lymph nodes is responsible for the late onset of post-surgical infections, where the lymph node channels are damaged and isolated. If presurgical translocation of bacteria does occur in vulvar cancer patients, should perioperative antibiotics be considered treatment rather than prophylaxis? Further studies to determine for which patients this might be a reasonable assumption remain to be done.

Second, the finding of a decreased incidence of wound infection following inguinal lymph node dissection when separate incisions were made complements previous work that has shown a decrease in the incidence of wound breakdown in these patients.[29] In addition, the sequence and time course of appearance of clinical infections following radical vulvectomy have been documented.

Finally, it is possible that, in large tumors, host immunity is decreased and musocal barriers are breached to a greater extent. It is important to determine if tumor stage and type increase the infectious morbidity of radical vulvar surgery. In this study, the frequency of wound infections was not directly related to tumor type or stage, but tended to increase only if inguinal lymph nodes were concomitantly removed. For example, if concurrent inguinal lymphadenectomy was performed, the incidence of wound infection was the same for stage I and stage III disease. Although the frequency of infection was evaluated in this investigation, the severity of infection was more difficult to quantify and compare. Importantly, no deaths from postoperative infections were found in this series.

Infectious morbidity associated with radical vulvectomy is an important clinical problem that deserves further investigation. Studies are difficult to execute by individuals, however, secondary to slow patient accrual and a wide variation in the surgical techniques employed. We hope that this work will stimulate interest among investigators so that prospective cooperative research will be forthcoming.

### REFERENCES

1. Carson LF, Barke RA, Brooker DC, Savage JE, Twiggs LB, Dunn DL: Infection and pelvic malignancies. In Carson CC III (ed): Problems in Urology. Vol 2. No 4. Philadephia: J.B. Lippincott, pp 499–510, 1988.
2. Brooker DC, Savage JE, Twiggs LB, Adcock LL, Prem KA, Sanders CC: Infectious morbidity in gynecologic cancer. Am J Obstet Gynecol 156:513–520, 1987.
3. Olm MJ, Galask RP: Bacterial flora of the cervix from two prehysterectomy patients. Am J Obstet Gynecol 122: 683–687, 1975.
4. Mead PB: Cervicovaginal flora of women with invasive cervical cancer. Obstet Gynecol 52:601–604, 1978.
5. Olm MJ, Scott JR, Galask RP: Cervical-vaginal flora of immunosuppressed renal transplant patients. Am J Obstet Gynecol 130:49–54, 1978.
6. Gorbach SL, Medna KB, Thadepalli H, et al.: Anaerobic microflora of the cervix in healthy women. Am J Obstet Gynecol 117:1053–1055, 1973.
7. Gilstrap LC, Gibbs RS, Michel TJ, et al.: Genital aerobic bacterial flora of women receiving radiotherapy for gynecologic malignancy. Gynecol Oncol 23:35–39, 1986.
8. Gerstner GJ, Kucera H, Weghaupt K, et al.: Endometrial bacteria in patients with endometrial cancer before and after primary intracavitary irradiation using IR-192 and an afterloading technique. Arch Gynecol 231:299–306, 1982.
9. Barber HRK: The critically ill patient with gynecologic cancer. Clin Obstet Gynecol 28:414–431, 1985.
10. Marsden DE, Cavanagh D, Wisniewski BJ, et al.: Factors affecting the incidence of infectious morbidity after radical hysterectomy. Am J Obstet Gynecol 152:817–821, 1985.
11. Mann WJ, Orr JW, Shingleton HM, et al.: Perioperative influences on infectious morbidity in radical hysterectomy. Gynecol Oncol 11:207–212, 1981.
12. Rosenshein NB, Ruth JC, Villar J, et al.: A prospective randomized study of doxycycline as a prophylactic antibiotic in patients undergoing radical hysterectomy. Gynecol Oncol 15:201–206, 1983.
13. Larsen B, Lifshitz S, Galask RP: Infectious diseases in gynecologic malignancy. In Buchsbaum NJ, Sciarra JJ (eds): Gynecologic Oncology. New York: Harper & Row, pp 1–11, 19, 1981.
14. Morgan LS, Daly JW, Monif GRG: Infectious morbidity associated with pelvic exenteration. Gynecol Oncol 10:318–328, 1983.
15. Scanlon EF: Infection following radical pelvic surgery. Gynecol Oncol 23:125–126, 1986.
16. Chambers JT, Kapp DS, Lawrence R, et al: Intermediate vs. delayed hysterectomy for endometrial carcinoma: Surgical morbidity and hospital stay. Obstet Gynecol 65: 245–250, 1985.
17. Hancock KC, Copeland LJ, Gershenson DM, et al.: Urinary conduits in gynecologic oncology. Obstet Gynecol 67:680–684, 1986.
18. Daly JW, Pomerance AJ: Groin dissection with prevention of tissue loss and postoperative infection. Obstet Gynecol 53:395–399, 1979.
19. Duff P, Gibbs RS: Pelvic vein thrombophlebitis: Diagnostic dilemma and therapeutic challenge. Obstet Gynecol Surv 38:365–373, 1983.
20. Fox LP: Fatal superinfection with monilia in gynecological surgery. Am J Obstet Gynecol 110:285–298, 1971.

21. Sweet RL, Yonekura ML, Hill G, et al.: Appropriate use of antibiotics in obstetrics and gynecologic infections. Am J Obstet Gynecol 146:719–739, 1983.
22. Cartwright PS, Pittaway DE, Jones HW III, et al.: The use of prophylactic antibiotics in obstetrics and gynecology: A review. Obstet Gynecol Surv 39:537–554, 1984.
23. Sevin B, Ramos R, Lichtinger M, et al.: Antibiotic prevention of infections complicating radical abdominal hysterectomy. Obstet Gynecol 64:539–545, 1984.
24. DiSaia PJ, Creasman WT: Clinical Gynecologic Oncology. 3rd ed. St. Louis: C.V. Mosby, p 258, 1989.
25. Sweet RL, Ledger WJ: Puerperal infections: A two year review. Am J Obstet Gynecol 117:1093–1100, 1973.
26. Stage AH, Long H, Silberman R, et al.: Wound infection following cesarean section. Surg Gynecol Obstet 145: 882–884, 1977.
27. Cruse, PJ, Foord R: A five year prospective study of 23, 649 surgical wounds. Arch Surg 107:206–210, 1973.
28. Sweet RL, Gibbs RS: Infectious Diseases of the Female Genital Tract. Baltimore: Williams & Wilkins, pp 155–160, 1985
29. Hacker NF, Leuchter RS, Berek JS, et al.: Radical vulvectomy and bilateral inguinal lymphadenectomy through separate groin incisions. Obstet Gynecol 58:574–579, 1981.
